# Retronychie: Operative Therapie und postoperative Versorgung: Retronychia: Surgical treatment and postoperative care

**DOI:** 10.1111/ddg.70202x

**Published:** 2026-11-10

**Authors:** Galina Balakirski, Alexander Kreuter, Silke C. Hofmann, Thorsten Neubert, Alexandros Paschos

**Affiliations:** ^1^ Zentrum für Dermatologie Allergologie und Dermatochirurgie Helios Universitätsklinikum Wuppertal Universität Witten/Herdecke Wuppertal Deutschland; ^2^ Klinik für Dermatologie Venerologie und Allergologie Helios St. Elisabeth Klinik Oberhausen Universität Witten/Herdecke Oberhausen Deutschland; ^3^ Klinik für Dermatologie Venerologie und Allergologie Helios St. Johannes Klinik Duisburg, Deutschland

**Keywords:** Avulsion, distaler Nagel‐Embedding, Fischmaulplastik, Kinesiologie Tape, Nagelchirurgie, Retronychie, Taping, avulsion, distal nail embedding, Howard‐Dubois technique, kinesiology tape, nail surgery, Retronychia, taping

## EINLEITUNG

Der Begriff Retronychie beschreibt das Einwachsen der proximalen Anteile der Nagelplatte in den proximalen Nagelfalz.^1^ Klinisch resultiert eine chronische Paronychie am proximalen Nagelfalz, eine Verdickung des proximalen Teils der Nagelplatte und eine Unterbrechung des Nagelwachstums. Typischerweise sind Großzehen betroffen, uni‐ oder bilateral. Pathogenetisch spielen ein zu langer Zehennagel mit konsekutivem Trauma, wiederholte Traumata oder Mikrotraumata, beispielsweise durch Sport oder Tragen unbequemer, enger Schuhe die wichtigste Rolle.^1^, ^2^, ^3^ Bei einem Trauma löst sich die Nagelplatte vollständig von der Nagelmatrix ab, eine neue Nagelplatte wächst unter der alten Nagelplatte, drückt diese nach oben und wird aber in die proximale Nagelfalz eingedrückt. So entstehen mehrere Nagelplattengenerationen, die bei Retronychie objektiviert werden.^1^, ^2^

Die genaue Prävalenz der Erkrankung ist unklar. Beschrieben wird, dass die Symptomatik häufig als bakterielle Infektion oder Mykose missdeutet wird und die Dauer bis zur Diagnosestellung und Therapie mehrere Monate bis Jahre betragen kann.^4^ Obwohl konservative Therapien wie Ablösung der hyperkeratotischen Nagelanteile mit harnstoffhaltigen Externa, podologische Behandlung mit Abschleifen der verdickten Nagelplatte bis zur letzten nagelbettnahen Nagelschicht, topische und intraläsionale Steroide sowie Anwendung von Tapeverbänden zur Linderung der Beschwerden führen können,^3^, ^5^, ^6^ ist die chirurgische Nagelavulsion von proximal die effektivste Therapieoption mit geringsten Rezidivraten (zwischen 10 und 21,8%).^7^, ^8^ Allerdings dauert das komplette Herauswachsen des neuen Zehennagels nach der Avulsion durch das physiologisch langsame Wachstum der Zehennägel mehrere Monate. Während dieser gesamten Zeit benötigt das Nagelbett eine konsequente Pflege um das Herauswachsen eines neuen, gesunden Nagels zu ermöglichen. Zudem kann das Aufwölben (Hypertrophie) des Hyponychiums bei fehlender Nagelplatte zum sogenannten „distalen Embedding*“* des Nagels, dem schmerzhaftem Einwachsen des distalen Nagelrandes in den distalen Nagelwall, führen. Somit erfordert die chirurgische Therapie der Retronychie für das optimale Endergebnis eine gute Mitarbeit der Betroffenen. Eine ausführliche Aufklärung der Patienten und regelmäßige postoperative Verlaufskontrollen sind bei der Therapie der Retronychie wichtig.

Nachfolgend zeigen wir am Beispiel zweier Retronychie‐Fälle mögliche postoperative Verläufe mit nachfolgender Versorgung und Therapieergebnissen.

## FALLVORSTELLUNG UND OPERATIONSTECHNIK

### Fall 1

Eine 39‐jährige Patientin litt seit etwa vier Monaten unter schmerzhafter Retronychie mit chronischer Paronychie und Granulationsgewebe am proximalen Nagelfalz des rechten Großzehennagels (Abbildung 1a). Einige Jahre zuvor wurde bei ihr wegen eines Unguis incarnatus des gleichen Großzehs eine sogenannte „Emmert‐Plastik“ durchgeführt. Wir führten eine minimalinvasive proximale Avulsion durch (Abbildung 1b). Es zeigte sich zu Beginn ein regelrechtes Nagelwachstum, wobei bei beginnender milder Hypertrophie des Hyponychiums bereits drei Monate nach Avulsion ein konsequentes Taping des distalen Nagelwalls begonnen wurde (Abbildung 1c). Allerdings kam es fünf Monate nach der Avulsion zum schmerzhaften distalen Embedding des Nagels (Abbildung 1d). Nach ausführlicher Aufklärung der Patientin wurde ein weiteres konservatives Vorgehen gewählt. Die Patientin führte täglich befeuchtende Nagelbettpflege fort und wandte Kinesiologie‐Tapes an, um die Hypertrophie des distalen Nagelwalls zu mildern (Abbildung 1e,f). Nach zwölf Monaten zeigte sich ein unauffälliger Nagelbefund (Abbildung 1g).

**ABBILDUNG 1:**
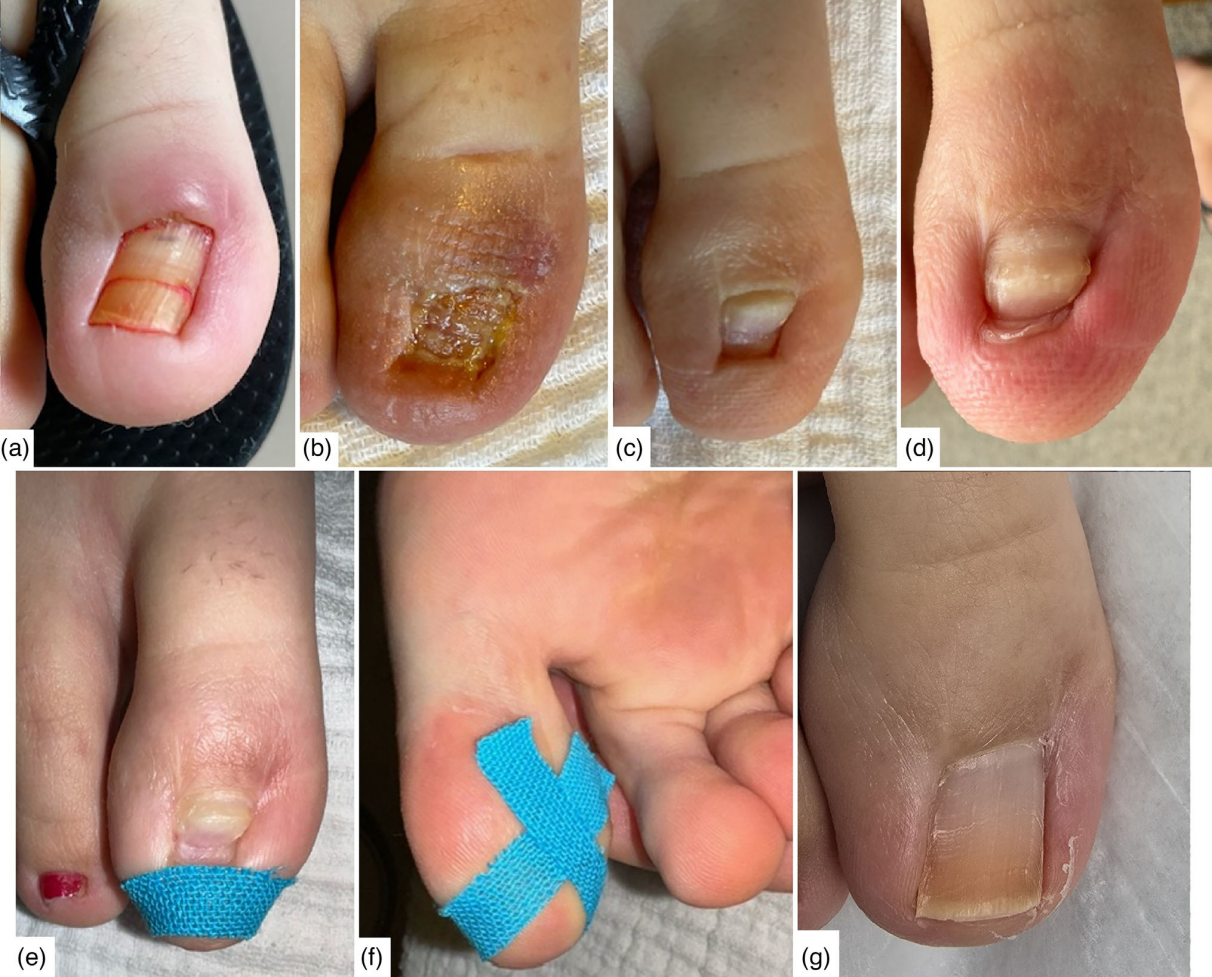
Klinische Präsentation der Retronychie mit Inflammation am proximalen Nagelfalz, Verfärbung des Nagels und mit sichtbaren reizlosen Narben nach Voroperation (a). Klinischer Befund zwei Tage nach Avulsion (b). Beginnendes Nagelwachstum drei Monate nach Avulsion mit einer bereits erkennbaren leichten Hypertrophie des distalen Nagelwalls (c). Deutliche Hypertrophie des distalen Nagelwalls mit beginnendem Einwachsen des distalen Nagelrandes fünf Monate nach Avulsion (d). Einsatz des Kinesiologie‐Tapes am distalen Nagelwall, um der Hypertrophie entgegenzuwirken, Ansicht von ventral (e) und von dorsal (f). Nagelbefund ein Jahr nach Avulsion mit regelrechtem Nagelwachstum (g).

### Fall 2

Eine 40‐jährige Patientin litt seit etwa fünf Monaten an einer schmerzhaften Retronychie mit chronischer Paronychie am proximalen Nagelfalz des linken Großzehs (Abbildung 2a). Es erfolgte eine minimalinvasive proximale Avulsion (Abbildung 2b). Zunächst wuchs der Nagel regelrecht (Abbildung 2c). Nach sechs Monaten kam es jedoch zum schmerzhaften Einwachsen des distalen Nagelrandes bei Hypertrophie des distalen Nagelwalls (Abbildung 2d). Die Anwendung der Kinesiologie‐Tapes lehnte die Patientin begründet durch Ungeduld ab. Um die Hypertrophie des distalen Nagelwalls zu beheben und das Herauswachsen des Nagels zu ermöglichen wurde eine sogenannte „Fischmaulplastik“ durchgeführt (Abbildung 2e,f). Bereits zwei bis drei Monate später zeigte sich ein unauffälliger Nagelbefund (Abbildung 2g).

**ABBILDUNG 2:**
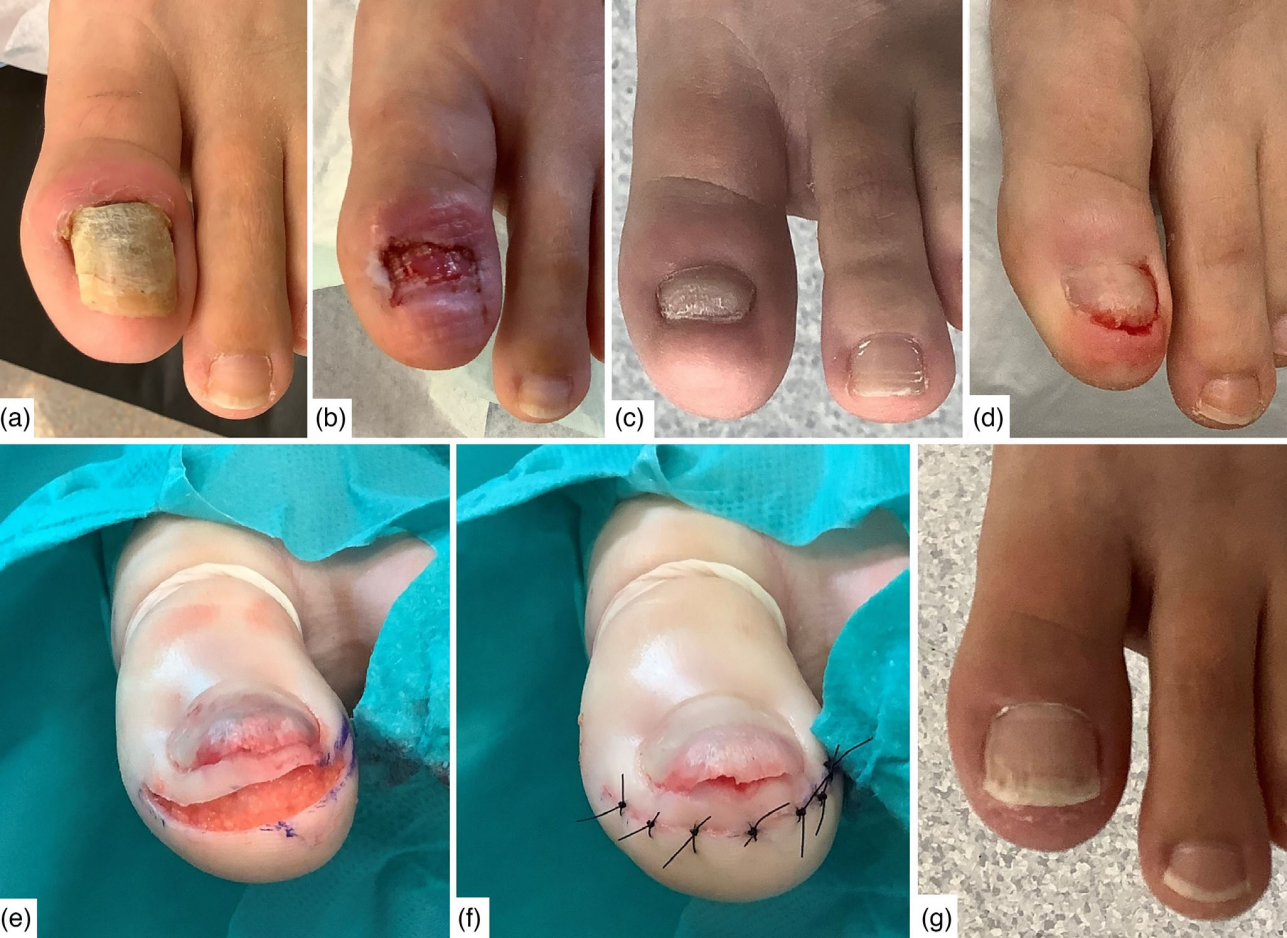
Klinische Präsentation der Retronychie mit Inflammation am proximalen Nagelfalz sowie Verdickung und Gelbfärbung des Großzehennagels (a). Klinischer Befund wenige Tage nach Avulsion (b). Beginnendes Nagelwachstum drei Monate nach Avulsion mit beginnender Hypertrophie des distalen Nagelwalls (c). Schmerzhaftes distales Einwachsen des distalen Nagelrandes bei deutlicher Hypertrophie des distalen Nagelwalls (d). Intraoperative Darstellung der „Fischmaulplastik“ zur Reduktion der Hypertrophie des distalen Nagelwalls mit der Schnittführung etwa 0,5 cm unterhalb des Hyponychiums (e) sowie Befund unmittelbar postoperativ (f). Nagelbefund zehn Monate nach Avulsion mit regelrechtem Nagelwachstum (g).

## DISKUSSION

Zurzeit existieren mehrere Fallberichte und Fallserien zu Therapien der Retronychie.^4^, ^5^, ^6^, ^7^, ^8^ Obwohl es Bemühungen gibt, eine effektive konservative Therapie der Retronychie zu etablieren, gilt die Avulsion von proximal weiterhin als Goldstandard und weist die niedrigste Rezidivrate auf.^9^ In einer prospektiven Studie wurden 20 Patienten mit Retronychie konservativ mittels Abfräsen des erkrankten Nagels bis zur letzten nagelbettnahen Nagelschicht behandelt.^6^ Die Sitzungen wurden alle 7 bis 14 Tage wiederholt, und im Falle einer Hypertrophie des distalen Nagelwalls erfolgten zusätzlich Tapeverbände. In allen Fällen kam es zur Abheilung der Nagelpathologie. Die Dauer der Therapie variierte dabei von 2 bis 14 Monaten, und nach insgesamt 18 Monaten zeigte sich kein einziges Rezidiv. Die Methode ist effektiv, allerdings auch verhältnismäßig aufwändig und sie benötigt mehrere Sitzungen.^6^ In einer multizentrischen Studie an 42 Patienten mit Retronychie war die Avulsion bei Retronychie mit einer Rezidivrate von etwa 10% der konservativen Therapie mit Therapieresistenz beziehungsweise Rezidivrate von 30% überlegen.^7^ Eine systematische Übersicht aus dem Jahr 2022 analysierte 231 Patienten aus insgesamt 24 Studien. Eine konservative Therapie führte in nur 41,2% der Fälle zur Abheilung, war allerdings sehr gut verträglich.^8^ Die Avulsion von proximal zeigte die besten Remissionsraten von 78,2%, war jedoch in 9,6% der Fälle mit langanhaltenden Komplikationen behaftet, meistens im Sinne von Nageldystrophien.^8^ Die Autoren schlossen, dass die konservative Therapie insbesondere bei milden Fällen einen hohen Stellenwert hat. Bei ausgeprägten Befunden wird jedoch eine Avulsion von proximal als Therapie der Wahl bevorzugt.^8^ Zu beachten ist allerdings, dass nach der Avulsion eine gute Patientenedukation und regelmäßige Verlaufskontrollen für den Therapieerfolg essenziell sind. Rehydrierende Nagelbettpflege und tägliches Massieren des Nagelbetts in Richtung des Nagelwachstums verhindern die Verhornung der nach der Avulsion ungeschützten, zarten Nagelbetthaut und verbessern die postoperativen Ergebnisse. Die Hypertrophie des distalen Nagelwalls kann durch Tapen überwunden werden (Abbildung 1e,f). In therapieresistenten Fällen oder bei unzureichender Patientenmitarbeit kann eine „Fischmaulplastik“ durchgeführt werden.^8^, ^10^ Unsere Fälle illustrieren diese beide Vorgehensweisen und betonen die Wichtigkeit der postoperativen Versorgung der Patienten mit Retronychie.

## INTERESSENKONFLIKT

Keiner.
